# What Are the Impacts of Introducing an SGLT2 Inhibitor after a Recent Episode of Acute Kidney Injury?

**DOI:** 10.34067/KID.0000000000000386

**Published:** 2024-03-26

**Authors:** Thyago Proença de Moraes

**Affiliations:** Postgraduate Program in Health and Biological Sciences—Pontifícia Universidade Católica do Paraná, Curitiba, Brazil

**Keywords:** Acute Kidney Injury, Chronic Renal Disease, Diabetic Nephropathy, SGLT2

The renoprotective effects of SGLT2 inhibitors were not expected when the first large controlled studies on cardiovascular safety emerged in the mid-2010s to test this new class of antidiabetic drugs.^1^ However, now they are considered a well-established and consolidated medication in controlling the progression of CKD in patients with or without diabetes.^2^ However, despite these excellent initial results at long term, concerns raised about the effect on a potential effect on AKI. This was mainly related to the glomerular hemodynamic effect of the class that leads to a characteristic drop in GFR during the first days after its introduction.^3^ Consequently, several groups turned their attention to evaluating data on AKI after the use of SGLT2 inhibitors. In another welcome surprise, a meta-analysis summarizing the results of 41 randomized clinical trials that pooled almost 70,000 patients demonstrated that not only was there no higher risk for AKI with SGLT2i but also participants randomized to SGLT2i actually had a markedly reduced risk of AKI compared with placebo.^4^

However, as the initial goal of the SGLT2 was solely glycemic control, the underlying mechanisms behind the new beneficial effect in the kidney were still unknown (and have not been completely elucidated yet). Some questions persistently trouble clinicians, raising apprehensions about their use in specific situations. Current guidelines, for example, suggests caution in the use of SGLT2i when there is a situation predisposing to AKI and that it should be temporarily suspended to avoid potential adverse events.^5,6^ However, there seems to be some understanding recently that SGLT2i may have a role even during an episode of AKI, and more importantly the delay in reintroducing SGLT2 inhibitors may be detrimental to the patient. Therefore, it is welcome when clinical data emerge confirming initial impressions and providing a certain reassurance to the usage while we do not have randomized controlled trials.

It is precisely in this literature gap that the study by Murphy and colleagues^7^ comes into play. Using data from the cohort of the largest integrated health care system in the United States, the Veterans Health Administration, the authors identified diabetic patients who, after January 2017, experienced a hospitalization event with AKI defined by a ≥50% increase in serum creatinine. The final follow-up time for the study was May 1, 2022. Inclusion criteria also involved the presence of proteinuria, which could have been identified by one of three distinct methods: an altered albumin-to-creatinine ratio (≥30 mg/g), a protein-to-creatinine ratio ≥150mg/g, or an altered urine dipstick (≥30). When a patient presented with more than one of these three tests, priority was given to the altered albumin-to-creatinine ratio, followed by the protein-to-creatinine ratio. The study had two primary outcomes, one aimed at assessing the progression of CKD post-AKI, and the other focused on the occurrence of a new AKI event. The study included more than 10,000 patients, of whom almost 2800 received an SGLT2 inhibitor after the index event. Among the outcomes studied, the recurrence of AKI occurred more frequently than the progression of CKD in the whole population as expected because of the shorter follow-up time.

In this study, the use of SGLT2 inhibitors reduced the risk of CKD progression by 28% and AKI recurrence by 25% in the adjusted models. The results are very welcome and reinforce the importance of SGLT2i in patients with DM and CKD, regardless of whether the patient has recently experienced an episode of AKI. The data from Murphy and colleagues^7^ add to and complement the recent and significant findings on the use of SGLT2i during an AKI episode in real-world patients.^8,9^

Despite the knowledge gained regarding the safety of SGLT2 inhibitors over the past few years, apprehension about their prescription still exists. These recently published studies in at-risk patients or those with established AKI are a first step toward expanding the use of this class of medications and reducing events that affect the patient's quality and duration of life, in addition to imposing a significant cost on the health care system. This behavior is interesting noted in the Veterans database where, on this study, only 20% of eligible patients in the database received an SGLT2 inhibitor post-AKI event.

Importantly, this is an observational study, and despite all the methodological care taken by the authors, there are significant limitations that deserve attention for a correct interpretation of the results. These limitations include a potential bias in the indication of SGLT2 inhibitors (propensity helps but does not eliminate the risk), the possible prescription of an SGLT2 inhibitor outside the capture by the VA system, and the necessity for the patient to have survived the first year post-AKI (survivor bias^10^). In addition, the results cannot be extrapolated to the subgroup of patients with GFR between 20 and 30 ml/min because they were not included in the study.

Despite the abovementioned limitations, the good study by Murphy and colleagues^7^ addresses a relevant and of interest of different fields. Its results lean toward supporting the maintenance/introduction of SGLT2 inhibitors shortly after an episode of AKI, serving as a prelude to ongoing controlled studies (NCT05360615) that will ultimately provide a definitive answer to the research question presented by Murphy *et al.*^7^
